# Growth of Ga_0.70_In_0.30_N/GaN Quantum-Wells on a ScAlMgO_4_ (0001) Substrate with an *Ex*-*Situ* Sputtered-AlN Buffer Layer

**DOI:** 10.3390/ma17010167

**Published:** 2023-12-28

**Authors:** Dong-Guang Zheng, Sangjin Min, Jiwon Kim, Dong-Pyo Han

**Affiliations:** 1 Department of Electronic and Communication, Hangzhou Dianzi University Information Engineering College, Hangzhou 311305, China; zdg@hziee.edu.cn; 2 Department of Photonics and Nanoelectronics, Hanyang University, Ansan 15588, Gyeonggi, Republic of Korea; sjimin75@hanyang.ac.kr (S.M.); kgw4755@hanyang.ac.kr (J.K.); 3 Department of Display & Semiconductor Engineering, School of Electrical Engineering, Pukyoung National University, Busan 48513, Republic of Korea

**Keywords:** quantum wells, metalorganic vapor phase epitaxy, screw-type dislocation, mixed-type dislocation, ScAlMgO_4_ (0001) substrate, sputtered-AlN buffer layer

## Abstract

This study attempted to improve the internal quantum efficiency (IQE) of 580 nm emitting Ga_0.70_In_0.30_N/GaN quantum-wells (QWs) through the replacement of a conventional *c*-sapphire substrate and an *in-situ* low-temperature GaN (LT-GaN) buffer layer with the ScAlMgO_4_ (0001) (SCAM) substrate and an *ex-situ* sputtered-AlN (sp-AlN) buffer layer, simultaneously. To this end, we initially tried to optimize the thickness of the sp-AlN buffer layer by investigating the properties/qualities of an undoped-GaN (u-GaN) template layer grown on the SCAM substrate with the sp-AlN buffer layer in terms of surface morphology, crystallographic orientation, and dislocation type/density. The experimental results showed that the crystallinity of the u-GaN layer grown on the SCAM substrate with the 30 nm thick sp-AlN buffer layer [GaN/sp-AlN(30 nm)/SCAM] was superior to that of the conventional u-GaN template layer grown on the *c*-sapphire substrate with an LT-GaN buffer layer (GaN/LT-GaN/FSS). Notably, the experimental results showed that the structural properties and crystallinity of GaN/sp-AlN(30 nm)/SCAM were considerably different from those of GaN/LT-GaN/FSS. Specifically, the edge-type dislocation density was approximately two orders of magnitude higher than the screw-/mixed-type dislocation density, i.e., the generation of screw-/mixed-type dislocation was suppressed through the replacement, unlike that of the GaN/LT-GaN/FSS. Next, to investigate the effect of replacement on the subsequent QW active layers, 580 nm emitting Ga_0.70_In_0.30_N/GaN QWs were grown on the u-GaN template layers. The IQEs of the samples were measured by means of temperature-dependent photoluminescence efficiency, and the results showed that the replacement improved the IQE at 300 K by approximately 1.8 times. We believe that the samples fabricated and described in the present study can provide a greater insight into future research directions for III-nitride light-emitting devices operating in yellow–red spectral regions.

## 1. Introduction

Since the first demonstration of a high-quality GaN layer on a sapphire substrate using a low-temperature (LT) buffer layer by Akasaki et al., there have been considerable advances in the research and development of AlGaInN-based electronic and optoelectronic semiconductor devices such as high-electron-mobility transistors, power devices, laser diodes, photodiodes, solar cells, and light-emitting devices (LEDs) [1,2,3]. In particular, white LEDs consisting of blue LEDs and yellow phosphor, which have now been widely adopted as general white lighting sources, are hailed as one of the most important scientific accomplishments of the twentieth century [4,5,6]. Moreover, white light sources consisting of three primary color LEDs can provide benefits to the human eye, including a color rendering index and circadian efficacy. Above all, they are attracting great attention nowadays to meet the global requirements for reducing carbon footprints thanks to their low power consumption, small size, and long lifetime span [7,8].

Despite the remarkable advances in AlGaInN growth technology over the last few decades, most modern III-nitride semiconductors are still fabricated on foreign substrates, i.e., *c*-sapphire (0001) substrates, due to the lack of economically acceptable free-standing GaN substrates [8,9]. For this reason, inherent problems caused by the adoption of *c*-sapphire substrates still pose significant challenges to researchers, including the existence of large differences in the lattice constants and thermal expansion coefficients (TECs) between the *c*-sapphire substrate and the GaN layer that cause a high dislocation density, wafer bowing, and large residual strain [10,11,12]. To overcome such inherent challenges, several foreign substrates other than *c*-sapphire substrates have been extensively investigated by several research groups, which include Si, SiC, GaAs, ZnO, *m*-sapphire, and *r*-sapphire substrates [12,13,14,15,16,17]. However, the crystallinity of GaN layers grown on such foreign substrates was found to be poor compared with that on *c*-sapphire substrates, and some substrates were not suitable for the modern growth technology of III-nitride semiconductors, i.e., metal-organic vapor-phase epitaxy (MOVPE) [14,15,16]. In particular, to the best of our knowledge, high-indium GaInN/GaN quantum wells (QWs) operating in the yellow–red spectral region exhibit very poor performance when employing the above-mentioned foreign substrates.

Regarding foreign substrates, a scandium magnesium aluminate [ScAlMgO_4_ (SCAM)] (0001) substrate has been intensively studied in recent years as a promising replacement for the *c*-sapphire substrate [18,19,20]. One motivation for the research on the SCAM substrate is that the *a*-lattice constant mismatch with GaN is as low as ~1.8%, while the mismatch with the *c*-sapphire substrate is as high as ~16% (the *a*-lattice constants of SCAM, sapphire, and GaN are 0.325 nm, 0.476 nm, and 0.319 nm, respectively) [18,19,20,21]. This similarity of *a*-lattice constants is expected to improve the crystal quality of the GaN layer, i.e., reduce the dislocation density. Additionally, the optical properties of SCAM are desirable for optoelectronic devices operating in the visible spectral region since they are transparent up to ~6.3 eV, and its refractive index is closer to that of GaN than that of sapphires (the refractive index of SCAM, sapphire, and GaN are 2.20, 1.77, and 2.56 in the visible range, respectively) [22]. Consequently, this similarity in refractive index is expected to reduce total internal reflection at the interfaces between the GaN layer and substrate. Most importantly, the coherent growth of the Ga_0.83_In_0.17_N layer on the SCAM substrate is possible in principle without a buffer layer since the *a*-lattice constant of the SCAM matches that of Ga_0.83_In_0.17_N [23,24]. Such unique properties are occasionally considered a key to realizing high-efficient III-nitride optoelectronic semiconductors operating in the yellow–red spectral region. Nevertheless, previous studies have shown that the improvement in the crystal quality and surface morphology of the Ga_0.83_In_0.17_N layer coherently grown on the SCAM substrate is not as good as expected [19,23,24]. This in turn indicates that a buffer layer is still required, even if Ga_0.83_In_0.17_N is grown on the *a*-lattice-matched SCAM substrate using MOVPE.

Previous studies on the buffer layer reported that an *ex-situ* sputtered-AlN (sp-AlN) buffer layer has great potential to improve the crystallinity of the GaN layer because it can reduce the growth time and thermal cycles of MOVPE growth [25,26,27]. In particular, the thermal properties of the AlN layer are desirable for the growth of high-quality GaN layer, i.e., the small difference in the TECs between the AlN layer and substrate are expected to reduce wafer bowing, dislocation density, and residual strain (the TECs of SCAM, sapphire, AlN, and GaN are ~6.2 × 10^−6^, ~8.3 × 10^−6^, 4.2 × 10^−6^, and 5.6 × 10^−6^/°C, respectively) [23,28]. Considering these advantages, improved crystallinity and reduced residual stress in an undoped-GaN (u-GaN) template layer grown on a *c*-sapphire substrate have been demonstrated by replacing a conventional in situ LT-GaN buffer layer with an *ex-situ* sp-AlN buffer layer [29]. Taking such improvements, an improved internal quantum efficiency (IQE) of green and ultraviolet-emitting QWs grown thereon has been demonstrated [27,29,30,31].

Aside from the above issues, recent studies have reported that the diffusion of Mg, Al, and Sc adatoms/atoms from the SCAM substrate into the GaN layer and the penetration of residual impurities (Mg, Al, and Sc) in the MOVPE reactor into the growth surface of the u-GaN layer during high-temperature growth are critical problems when the SCAM is employed as the substrate [21,23]. This is because Mg, Al, and Sc adatoms/atoms are easily separated from the SCAM at high temperatures due to their thermal instability and subsequently act as unwanted impurities in the GaN layer after diffusion/penetration. From this viewpoint, the employment of an sp-AlN buffer layer between the GaN layer and SCAM substrate could provide an additional advantage because the AlN layer can effectively suppress the diffusion of Mg, Al, and Sc adatoms/atoms from the SCAM substrate to the GaN layer during high-temperature growth owing to the role of the Al-rich layer in blocking diffusion of these impurities [23,32,33].

Collectively, considering the above-mentioned desirable properties of the SCAM substrate and the sp-AlN buffer layer for the growth of the GaN layer using MOVPE, one can expect that the crystal quality of the GaN layer and the IQE of QWs grown thereon would be significantly improved by simultaneously replacing a conventional *c*-sapphire substrate with a SCAM substrate and an LT-GaN buffer layer with an sp-AlN buffer layer, i.e., a GaN epitaxial layer grown on a SCAM substrate with an *ex-situ* sp-AlN buffer layer (GaN/sp-AlN/SCAM). However, to the best of our knowledge, this structure has not yet been demonstrated and reported.

In this context, the present study attempted to improve the crystal quality of a u-GaN template layer and enhance the IQE of Ga_0.70_In_0.30_N/GaN QWs grown thereon by replacing the conventional *c*-sapphire substrate and the buffer layer with the SCAM substrate and the sp-AlN buffer layer simultaneously. To this end, we first tried to optimize the thickness of the sp-AlN buffer layer by investigating the properties/qualities of the u-GaN template layer of the GaN/sp-AlN/SCAM in terms of surface morphology, crystallographic orientation, and dislocation type/density. After the optimization, we tried to grow Ga_0.70_In_0.30_N/GaN QWs on the GaN/sp-AlN/SCAM; the IQEs of QWs were measured by means of temperature-dependent photoluminescence (PL).

## 2. Preparation of the SCAM Substrate

**Figure 1:**
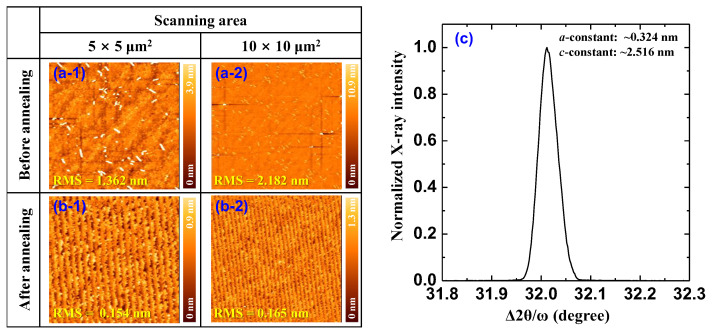
AFM images of the SCAM substrate (**a-1**,**a-2**) before and (**b-1**,**b-2**) after the thermal annealing process at 800 °C for 30 min for 5 × 5 and 10 × 10 μm^2^ scanning areas, and (**c**) 2θ/ω XRD scan spectrum of the SCAM substrate after the thermal annealing process.

**Figure 2:**
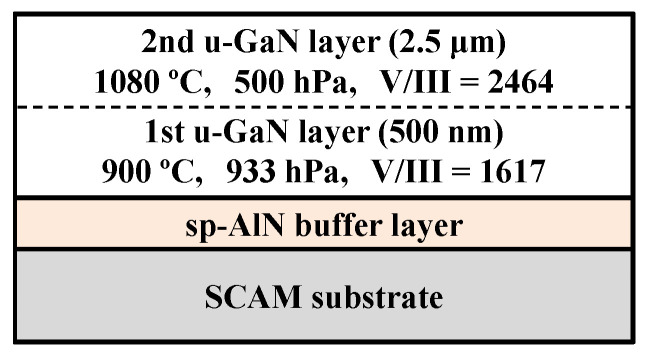
Schematic illustration of the sample (GaN/sp-AlN/SCAM) under investigation in this study.

As-received 430 μm thick SCAM substrates were thermally annealed to obtain a flat surface, i.e., to be suitable for modern MOVPE growth. Thermal annealing was performed in air at 800 °C for 30 min using an infrared rapid thermal annealing (RTA) system (RTA-4000, ULVAC Co., Chigasaki, Japan) [20,23]. Note that the SCAM substrate was found to deteriorate when the thermal annealing was performed at RTA temperatures above 800 °C due to the thermal instability of SCAM. After the thermal annealing process, the SCAM substrates were removed from the RTA system and examined via atomic force microscopy (AFM) and X-ray diffraction (XRD) measurements. Figure 1a,b show the AFM images of SCAM substrate before and after the thermal annealing process in two different scanning areas, which enabled us to confirm the effect of the thermal annealing process on the surface morphology. The results showed that the surface morphology of the SCAM substrate was significantly improved by the thermal annealing process. Specifically, the root-mean-squares (RMSs) of surface roughness decreased from 1.362 to 0.153 nm for a 5 × 5 μm^2^ scanning area (Figure 1(a-1,b-1)), and from 2.182 to 0.165 nm for a 10 × 10 μm^2^ scanning area (Figure 1(a-2,b-2)). We believe that the surface of the SCAM substrate after the thermal annealing process becomes atomically flat and thus appropriate for modern MOVPE growth [20]. The structural properties of the SCAM substrates after thermal annealing were evaluated via XRD measurements, as shown in Figure 1c. From the XRD data, the *a*- and *c*-lattice constants of the crystal were obtained and estimated as ~0.324 and ~2.516 nm, respectively [18,29]. Obviously, the structural properties of the SCAM substrate are in reasonable agreement with the above-mentioned theoretical predictions. That is, the *a*-lattice of the prepared SCAM substrate after the thermal annealing process closely matches that of Ga_0.83_In_0.17_N.

## 3. Optimization of the *Ex-Situ* sp-AlN Buffer Layer Thickness and Characterization of the GaN/sp-AlN/SCAM

An *ex-situ* sp-AlN buffer layer was deposited on the prepared substrate, i.e., the thermally annealed SCAM substrate, using a planar magnetron radio frequency (RF) sputtering system (CFS-4EP-LL, Shibaura Mechatronics Co., Yokohama, Japan). For the deposition, a sintered AlN target was placed approximately 85 mm from the SCAM substrate. After the chamber was evacuated to <5.5 × 10^5^ Pa, an Ar-N_2_ gas mixture was introduced into the system as the sputtering gas [27]. Using an RF power of 450 W, the sp-AlN layer was deposited on the SCAM at 500 °C. Note that the sp-AlN thin layer was deposited at a high temperature (500 °C is the highest deposition temperature of the sputtering system used in this study) to obtain a smooth surface [25]. In addition, the thickness of the sp-AlN layer was controlled by the deposition time, which is the optimization parameter in the present study. We will discuss this in detail in the following paragraph.

After the deposition of the sp-AlN buffer layer, the samples were loaded in an MOVPE reactor (EMC, Taiyo Nippon Sanso, Tokyo, Japan), and a 3 μm thick u-GaN layer was grown on the sp-AlN buffer layer. Figure 2 shows a schematic illustration of the prepared sample structure. The u-GaN layer consists of two layers, as shown in Figure 2. The first u-GaN layer was grown at a growth temperature of 900 °C and a reactor pressure of 933 hPa, with the precursors trimethylgallium (TMGa, flow rate = 20 sccm) and ammonia (NH_3_, flow rate = 3500 sccm), i.e., V/III = 1617, for a growth time of 30 min. The thickness of the first GaN layer was estimated to be 500 nm. The second u-GaN layer was grown at a growth temperature of 1080 °C and a reactor pressure of 500 hPa, with the precursors TMGa (flow rate = 45 sccm) and NH_3_ (flow rate = 12,000 sccm), i.e., V/III = 2464, for a growth time of 132 min. The thickness of the second GaN layer was estimated to be 2.5 μm. A two-step growth of the u-GaN layer was employed in this sample structure to promote two-dimensional lateral growth of u-GaN via nucleation and its coalescence processes [2,10]. In this study, the surface morphology, crystallographic orientation, and crystal quality of the prepared samples were compared with those of the conventional 3 μm thick u-GaN layer grown on flat *c*-sapphire with an in situ LT-GaN buffer layer sample (GaN/LT-GaN/FSS). Here, the conventional sample was prepared separately with a standard growth recipe [29].

**Figure 3:**
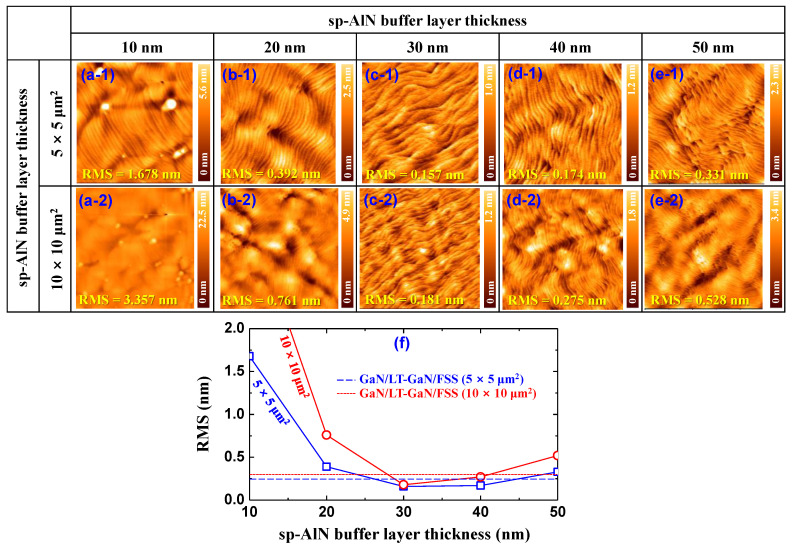
AFM images of the u-GaN surface of the GaN/sp-AlN/SCAM with various sp-AlN buffer layer thicknesses from 10 to 50 nm for (**a-1**–**e-1**) 5 × 5 and (**a-2**–**e-2**) 10 × 10 μm^2^ scanning areas. (**f**) RMS roughness plotted as a function of the sp-AlN buffer layer thickness for 5 × 5 and 10 × 10 μm^2^ scanning areas. The dashed lines represent the RMS roughness of the conventional GaN/LT-GaN/FSS.

We initially tried to optimize the thickness of the sp-AlN buffer layer. To this end, we prepared five samples with various thicknesses of sp-AlN buffer layers (ranging from 10 to 50 nm) by controlling the deposition time of RF sputtering. Note that the deposition and growth conditions of the sp-AlN buffer and u-GaN layers were identical for all samples except for the deposition time of the sp-AlN layer. To evaluate the samples, their surface morphologies were examined using AFM. Figure 3a–e shows the obtained AFM images and RMS roughness values of the u-GaN surface of the prepared sample in scanning areas of 5 × 5 and 10 × 10 μm^2^. Using these data, the RMS values were plotted as a function of the sp-AlN buffer layer thickness in Figure 3f for both scanning areas. The results showed that the RMS initially decreased and subsequently increased after reaching a minimum value at the sp-AlN thickness of 30 nm for both scanning areas. For the comparative study, the RMS of the conventional sample, i.e., GaN/LT-GaN/FSS, was also included therein (the dashed lines in Figure 3f). Findings revealed that the surface morphology of the u-GaN on the SCAM substrate with a 30 nm thick sp-AlN buffer layer [GaN/sp-AlN(30 nm)/SCAM] was superior to that of the GaN/LT-GaN/FSS.

**Figure 4:**
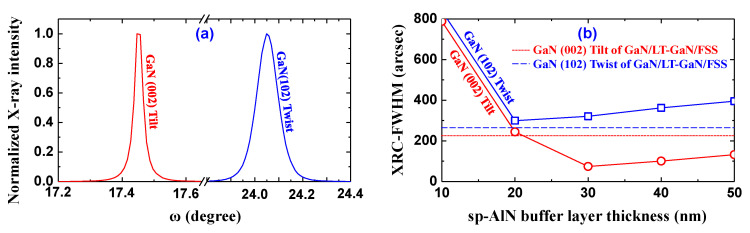
(**a**) Normalized XRC curves of GaN/sp-AlN(30 nm)/SCAM for the GaN (002) and GaN (102) planes. (**b**) XRC-FWHM of the GaN (002) and GaN (102) planes of the prepared samples plotted as a function of the sp-AlN buffer layer thickness. The dashed lines indicate the XRC-FWHM values of the GaN (002) and GaN (102) planes of the conventional GaN/LT-GaN/FSS.

Next, to characterize the distributions of the crystallographic orientations, we measured the X-ray rocking curve (XRC) and X-ray rocking curve-full width at half maximum (XRC-FWHM) of five samples since the XRC and XRC-FWHMs of the GaN (002) and GaN (102) planes represent the distribution of crystallographic orientations such as tilt and twist [34,35]. As representative examples, the XRC curves of GaN (002) and GaN (102) planes for GaN/sp-AlN(30 nm)/SCAM are plotted in Figure 4a. Likewise, the XRC-FWHM of GaN (002) and GaN (102) planes, i.e., tilt and twist, were plotted as a function of the sp-AlN buffer layer thickness in Figure 4b. Note that the dashed lines in Figure 4b indicate the XRC-FWHM values of the conventional GaN/LT-GaN/FSS. The GaN (002) plane in Figure 4b shows a similar sp-AlN buffer layer thickness-dependent characteristic with that of the surface morphology in Figure 3f, i.e., it initially decreases and then increases after reaching a minimum value at the sp-AlN layer thickness of 30 nm. Meanwhile, the GaN (102) plane exhibits different sp-AlN buffer layer thickness-dependent characteristics from those of the GaN (002) plane and surface morphology. More specifically, it reaches a minimum value at the sp-AlN thickness of 20 nm, and then increases slightly for a larger layer thickness. Considering the results in Figure 3f and Figure 4b, we speculated that the tilt orientation, i.e., GaN (002), is the main factor limiting the surface morphology over the twist orientation for the prepared GaN/sp-AlN/SCAM samples in this study. Moreover, the GaN (002) plane of the GaN/sp-AlN(30 nm)/SCAM is significantly smaller, and the GaN (102) plane is slightly larger than those of the GaN/LT-GaN/FSS, indicating a notable improvement in the tilt orientation. In contrast, there was no significant improvement in the twist orientation in the u-GaN layer.

Typically, the screw and/or mixed-type dislocations make a contribution to the broadening of the GaN (002) plane scan, and the edge- and/or complex-type dislocations contribute to the broadening of the GaN (102) plane scan [34,35]. That is to say, the simultaneous replacement of the LT-GaN buffer layer and the FSS with the 30 nm thick sp-AlN buffer layer and the SCAM substrate could greatly suppress the generation of the screw-/mixed-type dislocations (as opposed to the edge-type dislocation, which did not exhibit any significant changes). Notably, the edge-type dislocation density is estimated to be approximately two orders of magnitude higher than the screw-/mixed-type dislocation density in the GaN/sp-AlN(30 nm)/SCAM sample. Consequently, this replacement changed the dominant dislocation properties in the u-GaN layer.

**Figure 5:**
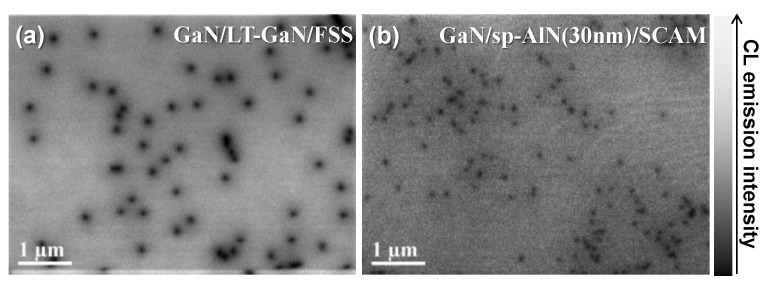
Plan-view CL mapping images taken with an acceleration voltage of 3 kV for (**a**) the GaN/LT-GaN/FSS and (**b**) GaN/sp-AlN(30 nm)/SCAM, respectively.

Next, to further investigate and compare the crystal quality of the prepared samples, plan-view cathodoluminescence (CL) mapping images were acquired using an acceleration voltage of 3 kV at room temperature, and the images are shown in Figure 5a,b for the GaN/LT-GaN/FSS and the GaN/sp-AlN(30 nm)/SCAM, respectively. The CL mapping image of GaN/LT-GaN/FSS in Figure 5a exhibited already well-known typical characteristics, i.e., a dislocation density and a dislocation diameter were estimated to be approximately 2.5 × 10^8^ cm^2^ and ~80 nm, respectively. Meanwhile, the CL mapping image in Figure 5b demonstrated different characteristics compared to the typical ones in terms of the following: (i) the overall CL image was slightly darker, and (ii) the dislocation diameter and density were smaller than those in Figure 5a. The differences in brightness between the CL images can be explained by the fact that the residual impurities in the MOVPE reactor (most likely Al, Mg, and/or Sc) separated from the SCAM substrate during the high temperature growth were penetrated into the growth surface, resulting in the intensive distribution of them at the u-GaN surface. After the penetration, the impurities act as shallow/deep levels in the forbidden gap, i.e., nonradiative recombination centers (NRCs), resulting in the decrease in CL emissions shown in Figure 5b [21,23,36]. In contrast, the different properties of the dislocation density and diameter between Figure 5a,b are believed to be due to the difference in the dominant type of dislocation between the samples. As explained in Figure 4b, the complex- and/or mixed-type dislocations are dominant in the GaN/LT-GaN/FSS, while the edge-type dislocation is dominant in the GaN/sp-AlN(30 nm)/SCAM. Therefore, all types of crystallographic orientation contributed to the deterioration of the crystal quality in GaN/LT-GaN/FSS, whereas the twist crystallographic orientation predominately contributed to the deterioration in the GaN/sp-AlN(30 nm)/SCAM sample. Thus, the crystallographic orientation seems to be the main factor limiting the dislocation properties of the u-GaN layer in this study. Consequently, the investigation of the samples at the initial stage of MOVPE growth would be informative to understand the overall characteristics in Figure 3, Figure 4 and Figure 5.

**Figure 6:**
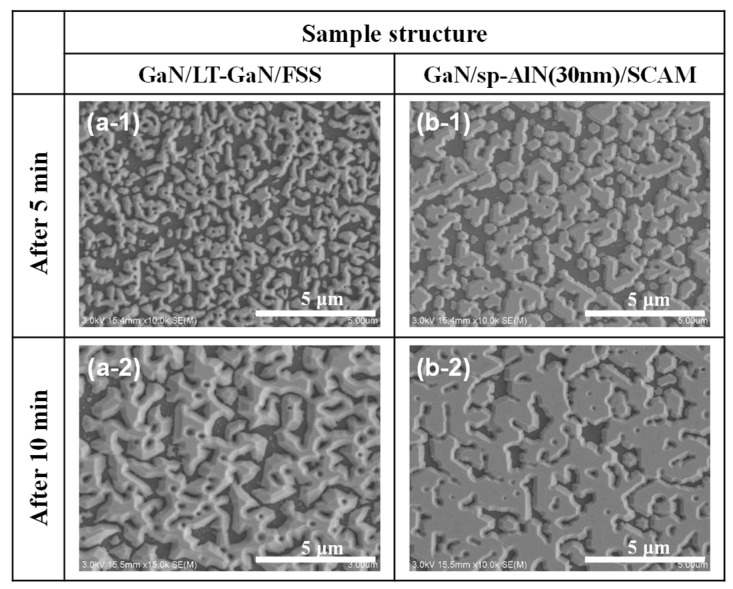
Plan-view scanning electron microscopy (SEM) images of (**a-1**,**a-2**) GaN/LT-GaN/FSS and (**b-1**,**b-2**) GaN/sp-AlN(30 nm)/SCAM taken after 5 and 10 min from initiating the growth of the u-GaN layer.

In an effort to understand the mechanisms behind the result shown in Figure 3, Figure 4 and Figure 5, we monitored the process of nucleation and its coalescence during the initial stage of MOVPE growth. Figure 6a,b show plan-view scanning electron microscopy (SEM) images of the GaN/LT-GaN/FSS and the GaN/sp-AlN(30 nm)/SCAM taken at 5 and 10 min after initiating the growth of the u-GaN layer. Significant differences in the initial growth between the two SEM images could be observed as follows: (i) the grain (island) size was larger, (ii) the aspect ratio of the grains was lower, and (iii) the island-island coalescence was greatly promoted in the GaN/sp-AlN(30 nm)/SCAM (Figure 6(b-1,b-2)) than those observed in the GaN/LT-GaN/FSS (Figure 6(a-1,a-2)). According to a previous study [37], the low aspect ratio (less than one) and large size of grains at the initial GaN growth typically introduce edge-type dislocations during the high-temperature island-island coalescence process. This is because dislocations besides the edge-type ones, i.e., screw-/mixed-type dislocations, are easily bent and annihilated during the high-temperature coalescence process. Furthermore, previous studies have identified that the promoted island-island coalescence process results in a decrease in dislocation density [37,38]. Consequently, we can see that the results presented in Figure 6 are consistent with the analysis of XRC-FWHM results in Figure 5, i.e., the GaN (102) plane is the predominant factor limiting the dislocation characteristics in the GaN/sp-AlN(30 nm)/SCAM, and lower dislocation density in the GaN/sp-AlN(30 nm)/SCAM (Figure 5).

Typically, the dislocation in the QWs is replicated from that of the lower layer, i.e., threading dislocation. Considering the results in Figure 5, one can expect a lower dislocation density in the QWs grown on the GaN/sp-AlN (30 nm)/SCAM rather than that grown on the GaN/LT-GaN/FSS. As a consequence, the performance of QWs subsequently grown on the GaN/sp-AlN (30 nm)/SCAM is expected to be improved since the dislocation characteristics of the u-GaN layer were changed due to the simultaneous replacement of the substrate and the buffer layer.

## 4. Growth and Evaluation of Ga_0.70_In_0.30_N/GaN QWs on SCAM Substrate with a 30 nm Thick *Ex-Situ* sp-AlN Buffer Layer

**Figure 7:**
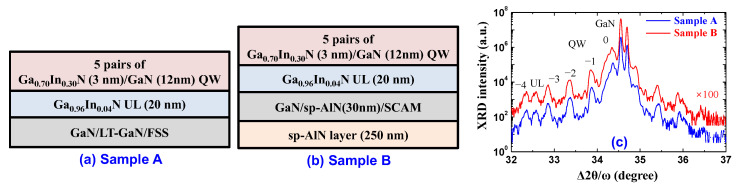
Schematic illustrations of the samples prepared in this study: the 20 nm thick Ga_0.96_In_0.04_N UL and the Ga_0.70_In_0.30_N/GaN QWs were sequentially grown on (**a**) GaN/LT-GaN/FSS (sample A) and (**b**) GaN/sp-AlN(30 nm)/SCAM (sample B). (**c**) Experimental results of the *2θ-ω* XRD scan spectra of samples A and B.

This section investigates the effects of the replacements on the subsequent QW active layers. For this purpose, the samples shown in Figure 7a,b were prepared. The QWs structure is identical for samples A and B: a 20 nm thick Ga_0.96_In_0.04_N underlying layer (UL) grown at 780 °C and five pairs of Ga_0.70_In_0.30_N (3 nm)/GaN (12 nm) QWs grown at 680 °C were sequentially stacked on the GaN/LT-GaN/FSS and GaN/sp-AlN (30 nm)/SCAM, respectively. Here, the In-containing UL between the u-GaN and QWs was inserted since the UL is believed to be able to trap/capture the defect at the u-GaN surface before its incorporation into the QWs [39,40]. It should be noted that a 250 nm thick AlN layer was deposited on the backside of the SCAM substrate (see Figure 7b) using the sputtering system before loading the SCAM substrate into the MOVPE reactor, which suppressed the separation of Mg, Al, and Sc adatoms/atoms from the SCAM substrate [23]. Consequently, the backside deposition of the AlN layer could reduce the penetration of residual impurities from the reactor into the growth surface. The deposition condition of the 250 nm thick AlN layer on the backside of the SCAM was identical to that of the sp-AlN buffer layer, with the exception of the deposition time.

The interfacial and structural properties of the QWs were investigated by measuring high-resolution *2θ*-*ω* XRD scan spectra for the prepared samples A and B. Figure 7c shows that the data were almost identical between the samples, including the separation of the peaks and the UL. This indicates that the effect of replacement on the interfacial and structural properties of the QWs was insignificant in the prepared samples.

**Figure 8:**
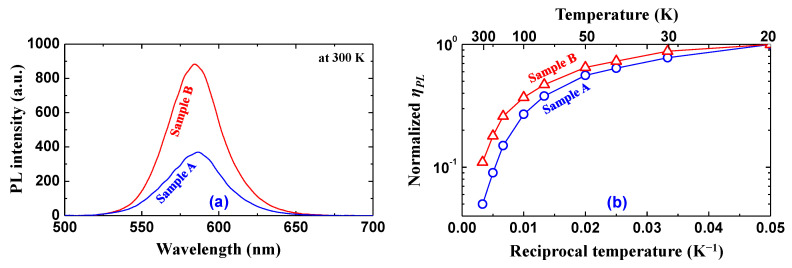
Experimental results of the (**a**) PL spectra at 300 K and (**b**) normalized temperature-dependent *η_PL_*, with an optical pumping source at 405 nm.

Next, to investigate the emission characteristics and performance of the QWs, we measured the PL spectra and PL efficiency (*η_PL_*) for samples A and B. We used a 405 nm continuous-wave semiconductor laser as the optical pumping source to excite the carriers only in the QWs [41]. Figure 8a shows the PL spectra of the samples at 300 K. The peak wavelengths were observed at around 580 nm for both samples, and the *η_PL_* of sample B seemed to be improved by approximately 1.8 times compared with that of sample A. The improvement of *η_PL_* in Figure 8a could be attributed to the improvement of IQE and/or light extraction efficiency (LEE). To clarify the origin of this improvement, we evaluated the IQE of both samples by measuring a temperature-dependent *η_PL_* (TDPL). This is a useful method in the qualitative evaluation of QWs because the NRCs are frozen out at cryogenic temperatures [42,43]. Figure 8b demonstrates the measurement results of the TDPL with a 405 nm optical pumping source for samples A and B. Therein, the data were normalized to each *η_PL_* at the lowest temperature (20 K in this experiment), i.e., each *η_PL_* at 20 K was assumed to have an IQE of unity. The IQEs at 300 K were estimated as 4.4% and 8.2% for samples A and B, respectively. This result indicates that the improvement of *η_PL_* at 300 K (Figure 8a) is mainly due to the improvement in IQE rather than the LEE. The observations in Figure 8 are consistent with our expectations in the analysis of Figure 5 and Figure 6, which suggested that the low dislocation density in the GaN/LT-GaN/FSS introduced a low dislocation density, i.e., a low NRC, in the QWs of sample B.

Furthermore, other than the high density of NRCs, the carrier localization in the QWs due to phase separation during low-temperature growth, the quantum-confined Stark effect in the QWs due to strain/stress, the defect-assisted Auger recombination, and the unbalanced carrier injection into the QWs from cladding layers due to the high activation energy of the acceptor are typically recognized as the causes of the low IQE of high-indium-content GaInN/GaN QWs [9,40,41,42,43,44,45]. However, we believe that they played an insignificant role in the samples under investigation in this study because the interfacial and structural properties of the QWs were almost the same for both samples (see Figure 7c), and the PL efficiency was investigated using quasi-resonant optical pumping [45,46,47]. Consequently, the reduced density and diameter of dislocations in the u-GaN template layer thanks to the replacement mainly contributed to an improvement in the IQE of the 580 nm emitting Ga_0.70_In_0.30_N/GaN QWs.

## 5. Summary

In summary, the present study examined the SCAM substrate with the *ex-situ* sp-AlN buffer layer to improve the crystallinity of the u-GaN layer and the QWs grown thereon. In an effort to optimize the sp-AlN buffer layers, they were deposited with various thicknesses (ranging from 10 to 50 nm) on the thermally annealed SCAM substrates by controlling the deposition time. The experimental results of AFM and XRC exhibited that the crystallinity of the u-GaN layer of GaN/sp-AlN(30 nm)/SCAM was superior to that of the conventional GaN/LT-GaN/FSS. Moreover, this comparative study revealed that the structural properties and crystallinity of the prepared sample were considerably different from those of the conventional samples, i.e., the edge-type dislocation density was approximately two orders of magnitude higher than the screw-/mixed-type dislocation density. To investigate the effect of replacement on the subsequent QW active layers, 580 nm emitting Ga_0.70_In_0.30_N/GaN QWs were grown on the u-GaN layers. The IQEs at 300 K were improved by approximately 1.8 times by the simultaneous replacement of the conventional substrate and the buffer layer with the SCAM substrate and the sp-AlN buffer layer. The results of IQEs were attributed to the suppression of the generated screw-/mixed-type dislocation. We believe that the samples fabricated and described in the present study can provide a greater insight into the future research directions for III-nitride LEDs operating in yellow–red spectral regions.

## Data Availability

The data presented in this study are available on request from the corresponding author. The data are not publicly available due to the project agreements.
